# Beneficial effects of loxapine on agitation and breathing patterns during weaning from mechanical ventilation

**DOI:** 10.1186/cc9015

**Published:** 2010-05-12

**Authors:** Benjamin Sztrymf, Guillaume Chevrel, Fabrice Bertrand, Dimitri Margetis, Dominique Hurel, Jean-Damien Ricard, Didier Dreyfuss

**Affiliations:** 1 Service de réanimation médicale, Hôpital Louis-Mourier, Assistance Publique-Hôpitaux de Paris, Université Denis Diderot, 178 rue des Renouillers 92701 Colombes Cedex, France

## Abstract

**Introduction:**

Interruption of sedation during weaning from mechanical ventilation often leads to patient agitation because of withdrawal syndrome. We tested the short-term efficacy and tolerance of loxapine in this situation.

**Methods:**

Nineteen mechanically ventilated patients with marked agitation after sedation withdrawal were included. Three agitation scales, the Richmond Agitation Sedation Scale (RASS), the Motor Activity Assessment Scale (MAAS), and the Ramsay and physiological variables (respiratory rate, airway occlusion pressure during the first 0.1 second of inspiration (P0.1), heart rate and systolic arterial blood pressure) were recorded before and after loxapine administration.

**Results:**

Loxapine dramatically improved all agitation scores (RASS and MASS decreased from 2 ± 0 to -1.1 ± 2.3, and 5.4 ± 0.5 to 2.7 ± 1.6, respectively; Ramsay increased from 1.0 ± 0 to 3.5 ± 1.5, 60 minutes after loxapine administration, *P *< 0.05 for all scores) as well as P0.1 (6 ± 4.2 to 1.8 ± 1.8 cm H_2_O; *P *< 0.05) and respiratory rate (from 31.2 ± 7.2 to 23.4 ± 7.8; *P *< 0.05) without hemodynamic adverse events. No side effects occurred. Sixteen (84%) patients were successfully managed with loxapine, sedation was resumed in two others, and one patient self-extubated without having to be reintubated.

**Conclusions:**

Loxapine was safe and effective in treating agitation in a small group of mechanically ventilated patients and improved respiratory physiologic parameters, enabling the weaning process to be pursued. A multicenter trial is under way to confirm these promising results.

## Introduction

ICU patients are constantly exposed to numerous nociceptive stimuli during their ICU stays. Most of them require appropriate sedation to maintain optimal levels of comfort and safety. This is particular true for patients with respiratory failure who require invasive mechanical ventilation to optimize mechanical ventilation and to avoid patient-ventilator asynchrony, especially during the acute phase of respiratory distress. Once this phase is over, efforts should be made to wean the patient from mechanical ventilation as fast as possible to reduce the length of invasive ventilation. Interruption of sedatives is an inevitable and necessary step in the weaning process. Weaning does require, however, full cooperation of the patient. The interruption of sedation at this period can lead to the patient's agitation with benzodiazepine or opioid withdrawal syndrome or both [1]. Risk factors for withdrawal syndrome, such as alcoholism, a history of hypertension, or the cumulative amount of sedative drugs have been identified [2]. Moreover, withdrawal reactions may be observed at the time of awakening in the setting of daily interruption of sedative infusion [3]. Agitation carries important proven side effects, such as increase in hospitalization duration [4], costs [5], long-term cognitive impairment, and mortality [6]. It has also been suggested that agitation could lead to weaning failure [7] and places patients at high risk of self extubation. No consensus exists concerning the management of agitation in this setting. Neuroleptic agents, such as haloperidol, have been proposed as the first-line drugs to administer in combination with nonpharmacologic procedures such as environmental control and psychological support [8]. Although this drug is used in some ICUs, it can induce drowsiness and decrease the patient's cooperation during the weaning procedure. In addition, extrapyramidal syndromes seem to occur more frequently after haloperidol than after other antipsychotic drugs [9-11]. Eventually, a large fraction of these patients are sedated again, leading finally to an increased period of invasive mechanical ventilation. Administration of loxapine, another neuroleptic agent, could be an interesting option in this setting, because of its good hemodynamic safety profile, its appropriate sedative properties, the rarity of its side effects, and its low cost. We, and those in many other ICUs in France, have been using this drug routinely for years to treat agitation [12], without ever precisely evaluating its sedative and physiological effects in this setting. Therefore we evaluated the short-term effects of loxapine on the agitation, breathing pattern, and hemodynamics in agitated patients during weaning from mechanical ventilation.

## Materials and methods

### Design and setting

This was a prospective single-center study in a university hospital intensive care unit.

### Patients

#### Inclusion criteria

Patients ventilated for >48 hours and considered potential candidates for weaning from the ventilator (resolution of the cause of acute respiratory failure, need for <50% FiO_2_, and <5 cm H_2_O positive end-expiratory pressure and hemodynamic stability according to SCCM weaning guidelines [13]) were prospectively monitored at the time of sedation (a combination of a benzodiazepine and an opiate) decrease or withdrawal. Patients were eligible for the study if they exhibited agitation after sedation decrease or removal, defined by RASS >1 [14].

#### Exclusion criteria

Patients with contraindications to the enteral administration of drugs were not eligible for the study. Patients with a known allergy to loxapine were excluded, along with epilepsy patients (because of the risk of convulsions associated with the use of neuroleptics).

### Study drug administration

A first enteral administration of 150 mg of loxapine was given via a nasogastric tube (that was already present for feeding or drug administration or both). If agitation (defined by a RASS >1) recurred within 90 minutes, a second administration of the same amount of loxapine was given. If the RASS remained >1 despite the cumulative dose of 300 mg, conventional sedation was resumed. The patient remained eligible for a new evaluation during the next attempt at sedation withdrawal.

### Data

Baseline demographic data, indication for and duration of mechanical ventilation, SAPS II [15], and the amounts of sedative agents given in the previous 24 hours were registered. The RASS and two other agitation scales were monitored: MAAS [16] and the Ramsay score [17]. We also monitored the following physiological variables: respiratory rate, heart rate, systolic arterial pressure, and airway occlusion pressure during the first 0.1 seconds of inspiration (P 0.1). This parameter was measured through the automated procedure available on the respirators used for this study (Evita 2 Dura, Evita 4, Evita Excel; Dräger, Lübeck, Germany) and provided an indication on the magnitude of respiratory-drive normalization provided by the study drug. All variables were monitored before the withdrawal of sedatives, at the time of agitation, and 60, 90, 120, and 180 minutes after initial administration of the study drug. Self-extubation or the unexpected self-removal of the nasogastric cannula or venous access were considered a failure of the drug, as was persistent agitation defined by an RASS score >1. The patients were closely screened for the following loxapine side effects: dyskinesia, extrapyramidal syndrome, seizure, neuroleptic malignant syndrome (defined by elevation of central temperature to >38°C, muscular rigidity, altered mental status, and autonomic dysfunction, such as unstable blood pressure or heart rate), and urinary retention.

### Ethical aspects

It has been usual practice for years in our ICU to administer loxapine to agitated mechanically ventilated patients. The protocol did not require any change in the dosage or the route of administration of the product. All measurements were strictly noninvasive, including the determination of P0.1, which was read from the respirator.

Consent could not be obtained from patients by definition, given their agitation. Consent was sought from proxies when they were present at the time of agitation. Otherwise, proxies were informed of all the procedures. Similarly, patients were informed of the details of the protocol as soon as their mental state allowed adequate comprehension. This protocol was approved by the Ethics Committee of the French Intensive Care Society (Société de Réanimation de Langue Française).

### Statistical analysis

Results are expressed as mean ± standard deviation. Changes over time of recorded variables were evaluated with one-way analysis of variance (ANOVA) for repeated measurements followed by Fisher's least significant difference test to detect differences between measurements. A difference was considered significant when *P *< 0.05.

## Results

Nineteen patients were included. Half of them had a history of chronic alcohol intake, as defined by drinking more than the equivalent of 1 L of wine per day for several years. Clinical characteristics, indication for mechanical ventilation, according to Zwillich *et al*. [18], are described in Table 1. The average dosage of midazolam and sufentanyl administered to the patients in the previous 24 hours before inclusion was noticeable (Table 1).

**Table 1:** Baseline characteristics of the patients

Age (years)	63.4 ± 13.2
Sex (m/f)	12/7
SAPS II	50 ± 9
Midazolam cumulative amount in the previous 24 hours (mg)	133 ± 128
Sufentanyl cumulative amount in the previous 24 hours (μg)	253 ± 225
Duration of MV (days)	10.4 ± 6.8
Indication for mechanical ventilation^a^	Acute respiratory failure (n = 7)
	Acute exacerbation of chronic respiratory impairment (n = 4)
	Toxic coma (n = 4)
	Sepsis (n = 3)
	Postoperative respiratory failure (n = 1)

MV, mechanical ventilation; SAPS, simplified acute physiology score; m, male; f, female.

^a^According to reference [14].

**Figure 1:**
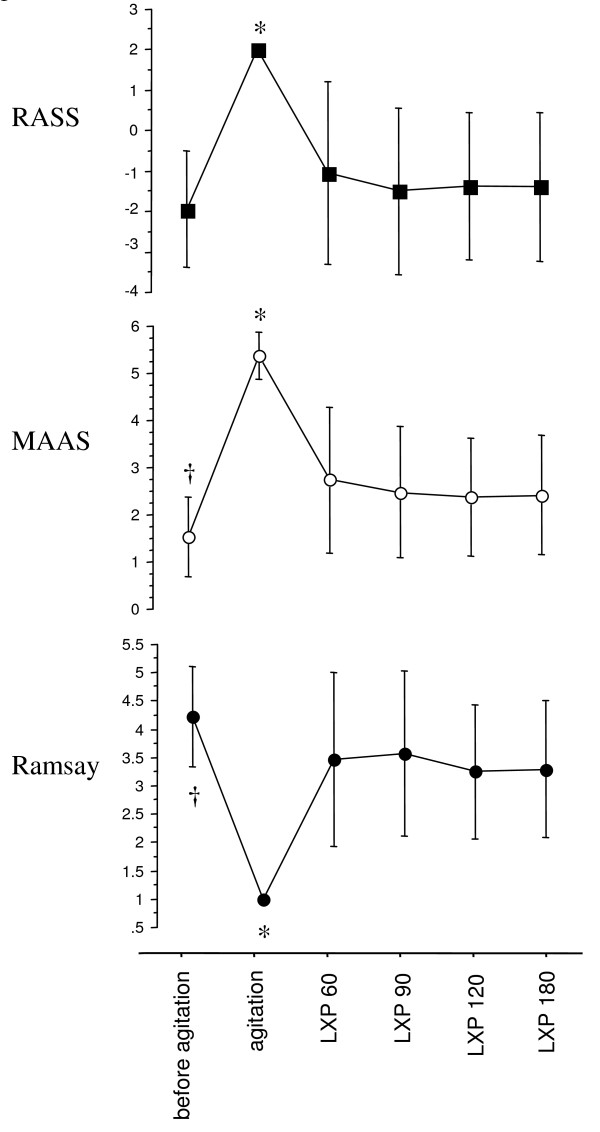
**Values for three sedation/agitation scores before agitation, at the time of agitation, and at 60, 90, 120, and 180 minutes after loxapine (LXP) administration**. Marked alteration of the three scores was initially observed. Loxapine administration resulted in normalization of the three scores after 1 hour. This normalization persisted for several hours. Significance of differences: *P < 0.0001 versus all other conditions; † *P *< 0.005 versus all other conditions.

**Figure 2:**
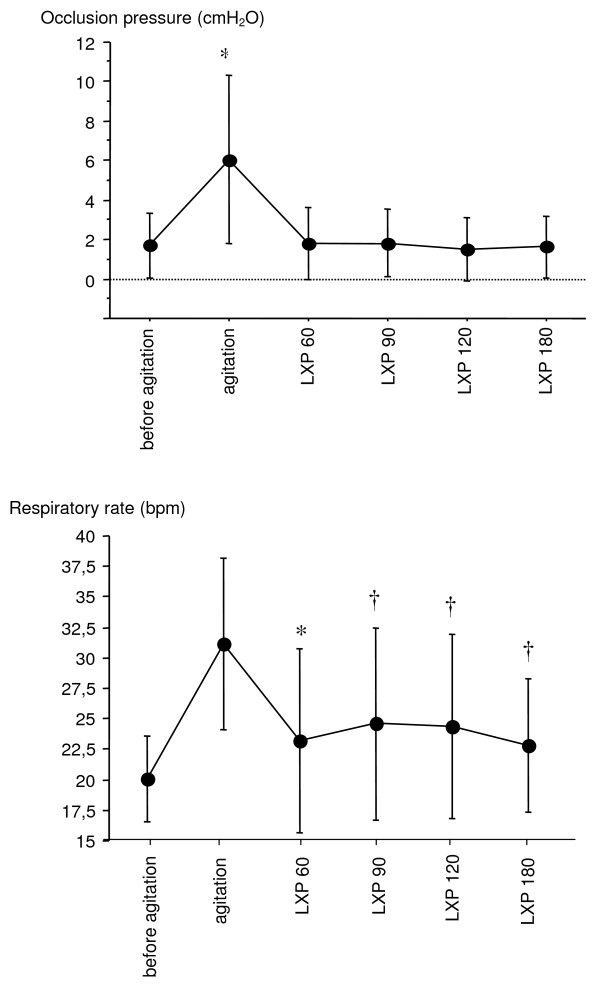
**Breathing-pattern scores before agitation, at the time of agitation, and at 60, 90, 120, and 180 minutes after loxapine (LXP) administration**. The dramatic increase in both respiratory rate and P0.1 observed during agitation normalized after loxapine administration. Significance of differences: *P < 0.0001 versus all other conditions; † *P *< 0.05 versus values observed before agitation.

Severe agitation was observed in the 19 patients after sedation withdrawal, as attested to by impressive changes in all three sedation/agitation scores, with marked increases in both MASS and RASS and a similar decrease in the Ramsay score (Figure 1). This agitation was accompanied by important increases in respiratory rate, P0.1, heart rate, and systemic systolic arterial blood pressure (Table 2 and Figure 2). All patients exhibited opioid or benzodiazepine withdrawal syndrome or both, as defined by Cammarano *et al*. [1].

**Table 2:** Effects of loxapine on hemodynamic parameters

	Before agitation	Agitation	60 minutes	90 minutes	120 minutes	180 minutes
HR (beats/min)	89.7 ± 15.6^a^	109.1 ± 19.4	108 ± 24.1	105.4 ± 24.2	108.9 ± 30.7	105.1 ± 25.3
SAP (mm Hg)	122.2 ± 22.1^b^	153.3 ± 29.3	136.8 ± 27.2	134.6 ± 26.2	136.8 ± 30.5	135.9 ± 27

^a^*P *< 0.0001 versus all other conditions.

^b^*P *< 0.005 versus value measured at the time of agitation.

HR, heart rate; SAP, systolic arterial pressure; beats/min, beats per minute.

Loxapine administration resulted in a dramatic improvement of agitation in 17 of the 19 patients. Failure of the drug occurred in two patients, despite the administration of two 150-mg doses of loxapine, as indicated in the protocol, and manifested as persistent agitation (RASS = 2; MAAS >5; and Ramsay = 1). These two patients required resumption of sedation to control the agitation and were excluded from further analysis. No agitation was observed when sedation was eventually discontinued, and both were successfully extubated after 2 and 4 additional days of ventilation, respectively. One of the 17 patients in whom loxapine administration had provided adequate control of agitation self-extubated 90 minutes after the first administration. This patient required no further ventilatory support and was discharged from the ICU 2 days later. Thus, 16 patients were available for analysis. The mean time between loxapine administration and efficacy, defined by a significant decrease in RASS, was 62 ± 39 minutes, during which, if necessary, gentle physical restraints (under medical supervision) and verbal reassuring were used while waiting for the drug to be effective. All values for the agitation/sedation scores were dramatically affected by loxapine administration, as attested to by a return to levels very close to those observed before agitation occurred (Figure 1). More precisely, values for RASS after loxapine administration were no longer different from those observed before agitation, whereas values for both MAAS and the Ramsay score were slightly, but significantly, different from those observed before agitation, indicating that patients were calm and cooperative but less sedated than before agitation occurred. The changes in respiratory pattern paralleled those of agitation sedation scores (Figure 2). Indeed, both P0.1 and respiratory rate dramatically decreased after loxapine administration to become no different from values observed before agitation for the former and only slightly higher for the latter. Heart rate and blood pressure decreased, but not significantly, after loxapine administration (Table 2).

No side effect of loxapine occurred in our cohort of patients.

## Discussion

Our study is the first to investigate the effects of loxapine administration in patients developing agitation after interruption of sedative drugs during weaning from invasive mechanical ventilation. We found that loxapine significantly reduced agitation in the vast majority of our patients, was well tolerated, and provided a calm and appropriate breathing pattern enabling the weaning process, instead of our having to resedate the patients.

Agitation is a common problem in the ICU and may result from many different causes, including anxiety, pain, delirium, withdrawal syndrome, shock, or respiratory distress. Because our patients' conditions had considerably improved when weaning was started, we believe that our patients were most likely agitated because of benzodiazepine or opioid withdrawal syndrome or both. Although we did not determine to what extent some may have experienced delirium, the fact remains that their mental status seriously compromised the weaning process. The scope of this study was not to provide a precise diagnosis for each encountered case but to study the effect of loxapine on agitation (after excluding that agitation was the result of a severe physical condition, that is, shock, pneumothorax, and so on). Agitation is a complex problem that may affect outcome. It has been shown that agitation *per se *is associated with a prolonged ICU stay, greater frequency of nosocomial infections, higher unplanned extubations, and central venous catheter removal rate, and a trend, although not significant, toward a higher mortality in one study [19,20]. It must be underlined that delirium was not monitored in these studies, and that some of the included patients might have exhibited agitation in the setting of delirium, with its proven negative impact on survival [6]. In particular, agitation impedes patient cooperation during weaning from mechanical ventilation and very often leads to delayed extubation. Undue prolongation of mechanical ventilation favors the occurrence of complications such as ventilator-associated pneumonia [21] or disuse atrophy of diaphragm [22] and increases hospital expenditures. First-line treatment for agitation consists mainly of nonpharmacologic interventions such as the establishment of a comfortable and reassuring environment, but this may not be sufficient in many instances. Sedative drug administration is thus often required to control agitation during weaning, but few studies have adequately addressed this issue [23]. Drugs aimed at controlling agitation during weaning should exhibit a rapid and sustained efficacy on neuropsychological disturbance with no or minimal impairment of both consciousness and respiratory drive, which would delay separation of the patient from the ventilator. Haloperidol is recommended by some authors but has numerous drawbacks, including extrapyramidal manifestations and significant QT_c _interval prolongation [9-11]. This drug was recently compared with a novel sedative and anxiolytic agent, dexmedetomidine, in agitated delirium [24]. In this open-label trial, dexmedetomidine was found to shorten median time to extubation and to reduce ICU length of stay in comparison with haloperidol. Frequent cardiovascular and hemodynamic side effects, such as bradycardia and hypotension, may, however, hinder the use of this promising agent [25]. Despite others' and our very long experience with loxapine, few if any prospective data exist on the use of loxapine in the ICU. In our preliminary clinical experience, as well as in that of others [12], loxapine characteristics and tolerance seem appropriate for use in this indication.

Our study clearly indicates that loxapine seems safe and efficient to treat agitation and allows a more physiologic breathing pattern during weaning from mechanical ventilation. This was attested to by normalization of three agitation/sedation scales, a dramatic improvement in the respiratory pattern, and excellent hemodynamic tolerance. Our three agitation and sedation scales describe awakening, anguish, agitation, and its subsequent threat of the removal of tubes or catheters. Before agitation, patients exhibited a state, described by RASS as 'light sedation, patients briefly awakened with eyes contact to voice,' by the Ramsay scale as 'patient with a brisk response to stimulus,' and by MAAS as 'patients responsive to touch or name with eyes opening or eyebrows raising or head turning when touched or name loudly spoken.' At the onset of agitation, patients exhibited typical agitation patterns such as 'anxious, restless, moving limbs out of the bed, fighting ventilator, attempting to sit up.' Loxapine administration led to interruption of this agitated state, with patients coming back to the previous 'light sedation status' or a cooperative state with 'sustained awakening, orientated and tranquil, following commands,' as described by the three scales. This dramatic improvement in sedation/agitation scores was paralleled by considerable decreases both in respiratory drive activity and respiratory rate, which returned to values similar to those observed in calm patients breathing spontaneously. Interestingly, these improvements were obtained without any hemodynamic deterioration, as indicated by stable heart rate and blood pressure (values for this latter parameter were no different from those measured before agitation occurred). No adverse reaction to loxapine was observed during this study.

Airway-occlusion pressure has been used for assessing output of the respiratory controller. It gives a measurement of a weighted sum of the effect of all respiratory muscles active at a given time and does not depend on the resistance or compliance of the respiratory system [26]. It has been suggested that this parameter is a sensitive and nonspecific marker of weaning failure [27], an elevated P0.1 meaning an increased inspiratory effort that might not be sustained. Respiratory-drive inhibition was never observed with 150 mg or in the two patients that required a cumulative amount of 300 mg of loxapine.

As discussed earlier, we observed that loxapine exerted its effects mainly on neuropsychic and respiratory disturbances, with few hemodynamic effects. Because withdrawal syndrome is characterized by sympathetic nervous system hyperactivity [28], adrenergic agonist agents like clonidine were used for that indication, with a certain degree of success [29]. It has been recognized that adrenergic agonists' effect in withdrawal syndrome is related to a decrease in sympathetic manifestations [28]. The targets of loxapine in the brain are dopamine and serotonin-receptor subtypes [30], with few hemodynamic effects, explaining why we observed no significant changes in hemodynamic parameters after loxapine administration. We hypothesize that the positive impact of loxapine on the agitation scores is related to its effect on anxiety and the observed decrease in respiratory rate.

This study is a preliminary physiological evaluation of the acute, short-term effects and safety of loxapine during weaning from mechanical ventilation in agitated patients. Several limitations of this study deserve consideration. First, loxapine did not allow adequate control of agitation in all patients: indeed, sedation was resumed in two patients because of persistent agitation despite two doses of loxapine. An additional patient was apparently calm after receiving loxapine but self-extubated during the study, which might also be considered as a failure of the drug. Nevertheless, loxapine was remarkably efficacious in the remaining 16 (84%) patients. The small number of patients in our study might have biased analysis of the potential side effects of loxapine. We emphasize, however, that we and others in France have been using loxapine for many years, without encountering noticeable side effects. It must be underlined that we did not specifically screen our patients for delirium. Therefore, although all our patients exhibited withdrawal syndrome, other reasons for agitation may have been present. The consequences of this last point on the interpretation of the results are unknown. Nonetheless, we were interested in evaluating the symptomatic effect of loxapine rather than investigating an etiologic treatment for agitation. In that respect, our results suggest that loxapine was effective in the vast majority of our patients.

## Conclusions

In conclusion, our study shows that loxapine seems to be safe and effective for treating acute agitation after withdrawal of sedative infusions during weaning from mechanical ventilation. It has a positive and sustained effect on several neurologic and respiratory disturbed parameters during withdrawal syndrome, enabling us to pursue the weaning process. Our results constitute the prerequisite for a randomized controlled study of the effects of loxapine on the duration of weaning in agitated mechanically ventilated patients. We are currently undertaking such a study.

## Key messages

• Agitation in the setting of withdrawal syndrome impedes patient cooperation during weaning from mechanical ventilation and often leads to delayed extubation.

• Loxapine has a positive effect on neurologic and respiratory disturbed parameters during withdrawal syndrome.

• Our study shows that loxapine seems to be safe and efficient for treating acute agitation during mechanical ventilation weaning after the withdrawal of sedative infusions.

• Further data are required to test the effect of loxapine on the duration of weaning.

## Abbreviations

HR: heart rate; ICU: intensive care unit; MAAS: Motor Activity Assessment Scale; RASS: Richmond Agitation Sedation Scale; RR: respiratory rate; SAP: systolic arterial pressure; SAPS II: simplified acute physiology score; SCCM: Society of Critical Care Medicine.

## Competing interests

The authors declare that they have no competing interests.

## Authors' contributions

BS, GC, JDR, DD made substantial contributions to the conception and design of the study. BS, GC, FB, DH, DM made substantial contributions to acquisition, analysis, and interpretation of the data. BS was involved in drafting the manuscript. JDR, DD revised the manuscript critically for important intellectual content and gave final approval of the version to be published.
